# A survey of the parasites of Ural saiga antelopes and Turkmenian kulans of Kazakhstan

**DOI:** 10.1016/j.ijppaw.2023.06.006

**Published:** 2023-06-20

**Authors:** Aida M. Abdybekova, Ainur A. Zhaksylykova, Kaissar Zh Kushaliyev, Erzhan Zh Kidiraliyev, Aigerim R. Kozhayeva, Ulbolsyn Zh Kuzhebayeva, Alexey Grachev, Alexandr Shevtsov, Christine M. Budke

**Affiliations:** a Kazakh Research Scientific Veterinary Institute, Almaty, Kazakhstan; b Zhangir Khan West Kazakhstan Agrarian-Technical University, Uralsk, Kazakhstan; c National Veterinary Reference Center, Almaty, Kazakhstan; d Institute of Zoology of the Republic of Kazakhstan, Almaty, Kazakhstan; e National Center for Biotechnology, Astana, Kazakhstan; f Texas A&M University, School of Veterinary Medicine & Biomedical Sciences, College Station, TX, USA

**Keywords:** Kazakhstan, Kulan, Helminths, Protozoa, Saiga

## Abstract

Saiga antelope and Turkmenian kulans are considered critically endangered and near threatened, respectively, by the International Union for Conservation of Nature (IUCN). Due to these species’ fragile status, it is important to understand the pathogens infecting their remaining populations. A total of 496 faecal samples were collected from Ural saiga antelope in western Kazakhstan during June, September, and November of 2021 and May and August of 2022 and 149 faecal samples were collected from kulans in the Altyn-Emel nature reserve in south-eastern Kazakhstan from June to August of 2021. Additionally, endo- and ecto-parasites were collected from 17 saiga that were found deceased due to natural causes. Nine helminths (3 cestodes, 6 nematodes) and two protozoans were found in Ural saiga antelope. In addition to intestinal parasites, one case of cystic echinococcosis due to *Echinococcus granulosus* infection and one case of cerebral coenurosis due to *Taenia multiceps* infection was identified on necropsy. None of the collected ticks (all *Hyalomma scupense*) were found positive for *Theileria annulate* (enolase gene) or *Babesia* spp. (18 S ribosomal RNA gene) via PCR. Three intestinal parasites (*Parascaris equorum*, *Strongylus* sp., and *Oxyuris equi*) were found in kulans. All identified parasites, in both saiga and kulans, are also found in domesticated livestock, suggesting a need for better understanding of how parasites are maintained within and between regional wild and domestic ungulate populations.

## Highlights

• Saigas and kulans have intestinal parasites also found in domestic livestock.
• Intestinal parasites infecting saigas and kulans appear to vary by season.
• The high proportion of saiga fecal samples containing *Eimeria* sp. is potentially concerning.

## Graphical abstract

**Figure:**
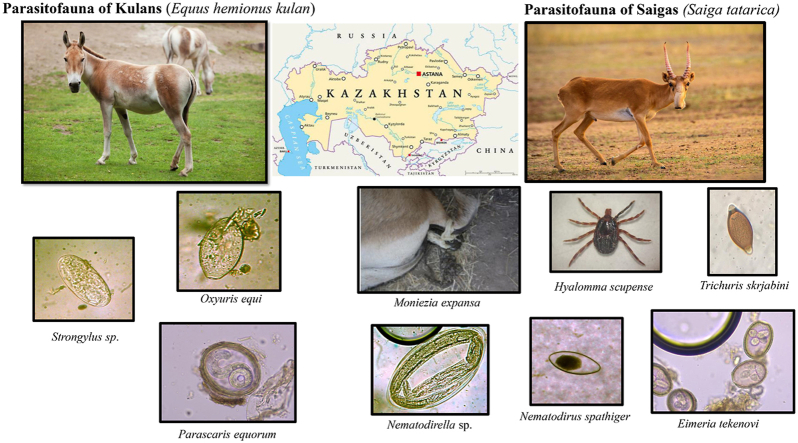

## 1 Introduction

There are approximately twenty species of wild ungulates that live in Kazakhstan, some having inhabited this region through antiquity (e.g., saiga antelope, Przewalski's horse, and kulan), while others are genetically related to modern domesticated livestock (e.g., mouflon, camels, argali, and wild boar). All of these species harbour a variety of protozoan and parasitic helminths and help maintain parasite lifecycles. For example, the elimination of cerebral coenurosis (caused by *Taenia multiceps*) in domestic sheep and goats is likely more difficult in regions where the parasite is also naturally reservoired in wild ungulates such as saiga or ibex (Kushaliev et al., 2022; Merbl et al., 2014; Moinet et al., 2010) Parasitic materials from wild ungulates from the temperate rangelands of Central Asia are scarce, with little research conducted over the last fifty years on the parasites of these animals. However, several recent research initiatives focusing on parasites of saiga antelope (*Saiga tatarica*) are encouraging (Karmaliyev et al., 2020; Akramova et al., 2020; Munib et al., 2022).

Saiga are considered critically endangered by the International Union for Conservation of Nature (IUCN) due to a drastic decline in numbers since the early 1990s (https://www.iucnredlist.org/species). In Kazakhstan, they are considered a protected species, with distribution ranges that are found in Kazakhstan, Mongolia, and Russia. The Ural saiga population is the largest in the world, numbering approximately 801,000 animals as of 2019. Efforts to protect this population from poaching and maintain a population size that allows for sustainability of the species are ongoing (Ministry of Education and Science of the Republic of Kazakhstan, 2019). Mass mortality events have occurred in this population, and parasitic diseases are believed to have been a contributing factor, at least to an event that occurred in 2010 (Kock, 2011).

Kulan (*Equus hemionus kulan*) are considered a near threatened (Category II) species by the IUCN. The modern distribution range, for this species, spans the deserts and semi-deserts of western and central Asia. In 1982, thirty-two (7 males, 20 females, and 5 foals) Turkmen kulan (*E. h. kulan,* Groves and Mazák, 1967) were brought to "Altyn-Emel" National Park from Barsakelmes Island. In 2022, there were 3611 kulan in the park. While the impact of many parasites on wild ungulates is unknown, some helminths and protozoans have been shown to cause death when an animal has a high parasite burden (Rubenstein and Hohmann, 1989; Debeffe et al., 2016). Therefore, it is important to understand which parasites are carried by these animals so that future studies can focus on their conservation and health impacts. The objective of this study was to evaluate the parasites of Ural saiga antelopes in western Kazakhstan and kulans in south-eastern Kazakhstan.

## 2 Materials and methods

**Fig. 1:**
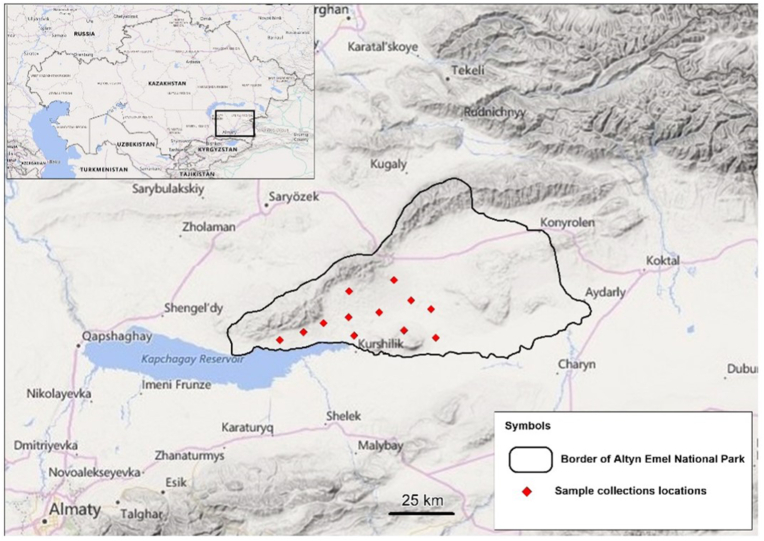
Map of Altyn-Emel National Park, with kulan faecal sample collection locations represented as red diamonds (June–August 2021). (For interpretation of the references to colour in this figure legend, the reader is referred to the Web version of this article.)

**Fig. 2:**
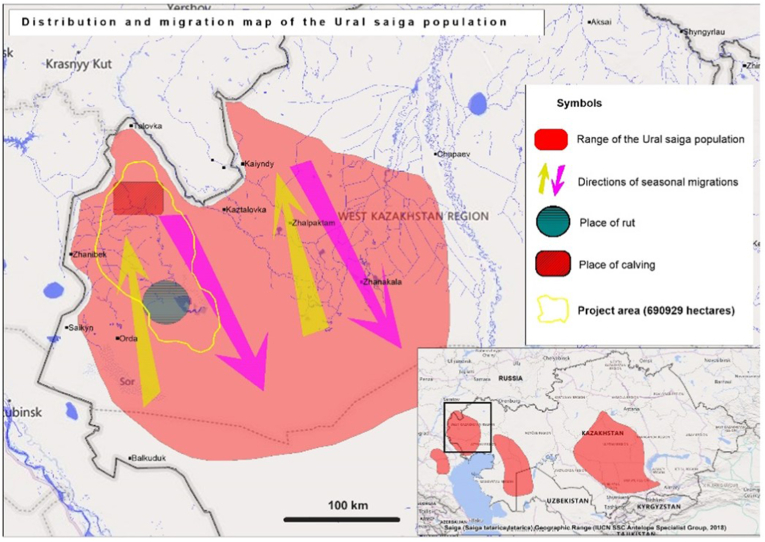
Map of the Ural saigas antelopes' distribution range, with study location enlarged. Arrows represent migration direction, while the blue circle represents rutting location, and the red square represents calving location. (For interpretation of the references to colour in this figure legend, the reader is referred to the Web version of this article.)

To determine what parasites were infecting saiga and kulan from Kazakhstan, faecal samples from Ural saiga antelope were collected from their native habitat in western Kazakhstan and faecal samples from kulans were collected from the State National Nature Park "Altyn-Emel" (SNPP "Altyn-Emel") in south-eastern Kazakhstan (Fig. 1). Naturally passed faecal samples were collected in spring, summer, and autumn of 2021–2022. Rangeland where saiga had recently been seen was searched for faecal samples (Fig. 2). Only those samples with a soft consistency were collected. Kulan faecal samples were collected around known water sources frequented by the animals. Faecal samples were attributed to saiga and kulan based on morphological characteristics with the aid of park rangers with extensive knowledge of these species. Faeces were collected in plastic containers with twist-on lids and transported to the parasitology laboratory of the Kazakh Research Scientific Veterinary Institute for further evaluation. Since samples were collected along migration routes, average transport time was ten to twelve days. Samples were kept on dry ice until they reached the laboratory where they were then refrigerated at 4 °C until evaluation.

All samples were evaluated using the Fülleborn and Darling methods. The Fülleborn method detects helminth eggs with a specific gravity up to 1.18, while the Darling method detects eggs with a specific gravity of 1.22–1.24. For the Darling method, 5–10 g of faeces were thoroughly mixed with water using a mortar and pestle. The resulting suspension was filtered through a metal sieve into centrifuge tubes and centrifuged for 2–5 min. The liquid was drained, and a mixture of equal parts glycerin and a saturated salt solution was poured into the precipitate. The contents of the test tube were thoroughly mixed and re-centrifuged. The liquid's upper film was removed with a platinum loop and transferred to a slide, covered with a glass coverslip, and examined under a microscope at 10x and 40x magnification. For the Fülleborn method, 3 g of faeces were placed in a polystyrene cup and 50–75 ml of saturated sodium chloride solution was gradually added while the suspension was stirred with a glass rod. The faecal suspension was filtered into another cup through a 0.3–0.5 mm metal strainer. After 45 min, the surface film was removed with a wire loop (diameter 0.8–1 cm) and placed on a slide. The material was then viewed under a microscope without using a coverslip at 10x and 40x magnification. Species identification of eggs and oocysts was performed according to previously described methods (Dunn and Keymer, 1986; Soulsby, 1982; Eckert et al., 1995; Heisin, 1967). Helminth eggs were evaluated for length, width, shape, colour, character of the outer shell, and internal structures following descriptions provided in two parasitology atlases (Kapustin, 1953; Svanbaev, 1979). No quantitative egg/oocyst counts were performed.

Seven saiga antelopes that died of unknown causes (2 females, 5 males) were examined in the summer of 2022 and ten additional deceased animals (3 females, 7 males) were necropsied in February 2023. Permission was granted by the Kazakh government to evaluate these deceased animals (permit number: KZ56VEP00127553). After a thorough evaluation of the tegument for external parasites, the body cavity was opened and the gastrointestinal tract and other organ systems, including the liver and brain, were evaluated grossly for evidence of parasite infection. Faecal samples were collected from the deceased animals and evaluated as described above. During the necropsy process, a muscle sample (60 g each) was collected from the diaphragm and leg of each carcass for trichinelloscopy according to the national protocol (Ministry of Agriculture, Order No. 7–1/393).

Collected ticks (n = 15) were evaluated for *Theileria annulate* (enolase gene) and *Babesia* spp. (18 S ribosomal RNA gene) via polymerase chain reaction (PCR) using the primers and protocol described by Kuibagarov et al. (2023) and Hilpertshauser et al. (2006). The remaining collected ticks (n = 2) were added to the Kazakh Scientific Research Institute's collection and were, therefore, not evaluated via PCR. Homogenization of ticks was carried out in a test tube containing 600 μL of viral transport medium (VTM) (Antigen, Kazakhstan) using 1.5 mm stainless steel balls and a Homogenizer Mixer Mill MM 400 (Retsch, Germany). The mixture was homogenized at 30 Hz for up to 6 times at 3-min intervals. DNA isolation from tick homogenates (100 μl) was carried out using the Ribo-Prep Nucleic Acid Isolation Kit (Cat. K2-9-Et-100, AmpliSens, Russia). The PCR reaction contained 12.5 μL of BioMaster HS-Taq PCR (2×) (Biolabmix, Russia), 1 μL of each primer pair at 10 pM/μL, 8 μL of DNA, and nuclease-free water to achieve a final reaction volume of 25 μL. Reactions were run on a SimpliAmp™ Thermal Cycler (Applied Biosystems™).

## 3 Results

In total, 496 faecal samples from Ural saiga antelopes were collected and evaluated. In June 2021, the overall infection prevalence was 69.31% (70/101), with five helminths and one protozoan identified (Table 1). Fifty-seven (81.43%) of the infected saiga were infected with only one intestinal parasite, while thirteen (18.57%) where infected with more than one parasite. In September 2021, 95 faecal samples were collected, and four helminths and two protozoans were identified (Table 1). The overall infection prevalence was 17.89% (17/95). Thirteen animals (76.47%) were infected with a single parasite, while four (23.53%) were co-infected. In November 2021, 105 saiga faecal samples were collected and examined, but no helminth eggs or *Eimeria* oocysts were found. In May 2022, 53 faecal samples were collected and examined, with again no helminth eggs or *Eimeria* oocysts identified. Finally, in August 2022, 142 saiga faecal samples were examined, of which one sample (0.70%) contained *Moniezia expansa* eggs, one sample (0.70%) contained *Nematodirella* sp. eggs, and 31 samples (21.83%) were positive for *Eimeria elegans* oocysts (Table 1).

**Table 1:** Saiga faecal sample findings (June 2021–August 2022).

Parasite	June 2021	September 2021	November 2021	May 2022	August 2022	Overall
*Oesophagostomum venulosum*	6/101 (5.94%)	3/95 (3.15%)	0/105 (0%)	0/53 (0%)	0/142 (0%)	9/496 (1.81%)
*Trichuris skrjabini*	3/101 (2.97%)	0/95 (0%)	0/105 (0%)	0/53 (0%)	0/142 (0%)	3/496 (0.60%)
*Nematodirus spathiger*	6/101 (5.94%)	2/95 (2.10%)	0/105 (0%)	0/53 (0%)	0/142 (0%)	7/496 (1.41%)
Nematodirella sp.	6/101 (5.94%)	2/95 (2.10%)	0/105 (0%)	0/53 (0%)	1/142 (0.70%)	9/496 (1.92%)
*Moniezia expansa*	3/101 (2.97%)	0/95 (0%)	0/105 (0%)	0/53 (0%)	1/142 (0.70%)	4/496 (0.81%)
*Eimeria elegans*	62/101 (61.38%)	12/95 (12.63%)	0/105 (0%)	0/53 (0%)	31/142 (21.83%)	105/496 (21.17%)
*Eimeria tekenovi*	0/101 (0%)	2/95 (2.10%)	0/105 (0%)	0/53 (0%)	0/142 (0%)	2/496 (0.40%)

Partial necropsies were performed in the summer of 2022 on two female and five male yearling saiga antelope that died of natural causes. Six of the animals (87.71%) were infected with adult intestinal helminths, with *M. expansa* found in 5 saigas, *Nematodirus spathiger* found in six animals, and *Trichuris ovis* found in 4 saigas. The larval stage of *Echinococcus granulosus* was found in the liver of one animal and the larval stage to *T. multiceps* was found in the brain of one animal. One of the necropsied animals was only infected with *M. expansa* (12 adult tapeworms found), while the other five parasitized animals were infected with more than one parasite genus. Evaluation of the muscle showed no indication of tapeworm larvae or trichinellosis.

In February 2023, necropsies were performed on ten additional Ural saiga (3 females less than 1 year of age and 7 males aged 1–3 years) found dead due to natural causes. During this evaluation, no parasites were found in the gastrointestinal tracts (including on faecal examination), livers, lungs, muscles, or brains of any of the evaluated animals. Mites were found on the abdomen and neck of two of the evaluated saigas (1 female, 1 male) and 2 ticks (*H. scupense*) were found on the neck of one of the females and 15 *H. scupense* ticks were found on the abdomen of one of the males. No PCR products characteristic of *Theileria annulata* or *Babesia* spp. were amplified from the evaluated ticks.

In total, 149 kulan faecal samples were collected from June to August 2021. Intestinal parasites were identified in twenty-five (16.78%) of the samples and included, *Parascaris equorum* (n = 7; 28.00%), *Strongylus* sp. (n = 19; 76.00%), and *Oxyuris equi* (n = 1; 4.00%). None of the samples contained more than one parasite type.

## 4 Discussion

The distribution range for saiga antelope and kulan is limited largely to the steppes of Kazakhstan and Mongolia. Due to the precarious nature of this ecosystem, it is important to help ensure the health of the inhabiting species, including wild ungulates. While parasites are a critical part of any ecosystem, they can be harmful especially if the infected animals are under stress. Therefore, it is important to understand the parasites infecting endangered species and incorporate this information with other conservation practices.

Species composition of saiga parasites appears to have decreased over the last 15–20 years, possibly due to external factors such as climate change. For example, in the 1990s, 36 parasite species (5 cestodes, 24 nematodes, and 7 protozoans) were found in the Ural saiga population (Bekenov et al., 1998) and, in the early 2000s, Morgan et al. (2005) found 15 species (3 cestodes and 12 nematodes). In comparison, the current study only identified nine helminths (3 cestodes, 6 nematodes) and two protozoans in Ural saiga antelope. In the current study, differences in intestinal parasite presence were found between sampling dates. These differences could be due to changes in diet or variations in the presence of various intermediate or reservoir hosts. It should be noted that a limitation of this study is that all parasitic material was acquired by convenience sampling. Therefore, intestinal parasite prevalence cannot be readily extrapolated to the entire populations of saiga or kulan in the study areas. In addition, the relatively small number of samples should be taken into consideration when interpreting study findings. While park rangers with expertise in wild ungulate management provided identification of faecal sources, another limitation of this study is that samples were not genetically tested, which could potentially lead to misidentification.

All ticks collected from saiga were *H. scupense.* A recent study identified *H. scupense* as the fifth most common tick found on livestock sampled from throughout Kazakhstan (Sultankulova et al., 2022). While *T. annulate* was not identified in the ticks evaluated as part of the current study, this pathogen has been isolated from *H. scupense* ticks attached to regional cattle (Sultankulova et al., 2022). Therefore, it is important to monitor wild ungulates for this protozoan. Similarly, while *Babesia* spp. was not identified in the evaluated ticks, *Babesia caballi* has been amplified in *H. scupense* ticks collected from horses in Kazakhstan (Sultankulova et al., 2022).

Four helminths were identified in kulans, all of which are also found in equids. Parasite composition, in the current study, was similar to that found during a study of kulans conducted on the former island of Barsa-Kelmes, where *Parascaris equorum* and *Strongylus* sp. were identified (Baitursinov, 2008). Infection with *Strongylus* sp. is believed to be common in kulans due to the relatively large cecum volume, where this parasite species is localized. In 2017–2018, *Oxyuris equi* eggs and large and small strongyles (Subfamilies Strongylinae and Cyathostominae) were identified in the faeces of seven of nine Turkmen kulans (78%) that were transported more than 1300 km from the Altyn-Emel to the central steppes as part of a reintroduction project (Gliga et al., 2020).

All helminths and protozoa identified in the current study are also pathogenic to domestic livestock. The high infection prevalence with *Eimeria* spp. (13.68%, 13/95), including one sample infected with both *Eimeria elegans* and *Eimeria tekenovi*, is especially worrisome since heavy *Eimeria* infections have been linked to mortality in young livestock (Chandra Deb et al., 2022). High intensity infections with *Eimeria* spp. can result in anorexia, diarrhoea containing blood and mucus, and lethargy. More chronic infections can lead to weight loss and development issues in young animals. Similarly, animals infected with *Moniezia* spp. tend to quickly lose weight and intestinal blockage and possibly intestinal rupture can occur with heavy parasite loads (Diop et al., 2015; Liu et al., 2019; Abdelhamid et al., 2021). Hepatic, pancreatic, and intestinal disorders have also been attributed to *Moniezia* infection (Kushaliyev et al., 2023).

Mature *Nematodirus* feed on the host's blood, causing anaemia, lethargy, and decreased growth. Acute diarrhoea associated with this parasite can also lead to death in young animals. One of the necropsied saiga had cerebral coenurosis due to *T. multiceps*. While this was not definitively determined to be the cause of death for this animal, young animals can die due to this condition. In general, helminths and protozoal infections can impact the immune system, making animals more susceptible to secondary infections (Mulcahy et al., 2004). While it is believed that this is also true for saiga antelope, there have been no observational or experimental studies demonstrating this relationship in saiga, although secondary infections with *Pasteurella* have been anecdotally reported (Orynbayev et al., 2019).

In conclusion, this study provided information on the current composition of parasites of two at risk species, the Ural saiga antelope in western Kazakhstan and the Turkmen kulan in south-eastern Kazakhstan. Since the identified parasites are also common in domesticated livestock, additional research is needed to better understand how parasites are maintained within and between regional wild and domestic ungulate populations.

## Declaration of competing interest

Conflict of Interest None.
